# Consumer Adoption of Future MyData-Based Preventive eHealth Services: An Acceptance Model and Survey Study

**DOI:** 10.2196/jmir.7821

**Published:** 2017-12-22

**Authors:** Timo Koivumäki, Saara Pekkarinen, Minna Lappi, Jere Väisänen, Jouni Juntunen, Minna Pikkarainen

**Affiliations:** ^1^ Martti Ahtisaari Institute Oulu Business School University of Oulu Oulu Finland; ^2^ VTT Technical Research Centre of Finland Oulu Finland; ^3^ Department of Marketing Oulu Business School University of Oulu Oulu Finland; ^4^ Research Unit of Medical Imaging, Physics and Technology Faculty of Medicine University of Oulu Oulu Finland

**Keywords:** health behavior, consumer behavior, eHealth, surveys and questionnaires, personal health record, patient-accessible health record, adoption, UTAUT, PHR

## Abstract

**Background:**

Constantly increasing health care costs have led countries and health care providers to the point where health care systems must be reinvented. Consequently, electronic health (eHealth) has recently received a great deal of attention in social sciences in the domain of Internet studies. However, only a fraction of these studies focuses on the acceptability of eHealth, making consumers’ subjective evaluation an understudied field. This study will address this gap by focusing on the acceptance of MyData-based preventive eHealth services from the consumer point of view. We are adopting the term "MyData", which according to a White Paper of the Finnish Ministry of Transport and Communication refers to "1) a new approach, a paradigm shift in personal data management and processing that seeks to transform the current organization centric system to a human centric system, 2) to personal data as a resource that the individual can access and control."

**Objective:**

The aim of this study was to investigate what factors influence consumers’ intentions to use a MyData-based preventive eHealth service before use.

**Methods:**

We applied a new adoption model combining Venkatesh’s unified theory of acceptance and use of technology 2 (UTAUT2) in a consumer context and three constructs from health behavior theories, namely threat appraisals, self-efficacy, and perceived barriers. To test the research model, we applied structural equation modeling (SEM) with Mplus software, version 7.4. A Web-based survey was administered. We collected 855 responses.

**Results:**

We first applied traditional SEM for the research model, which was not statistically significant. We then tested for possible heterogeneity in the data by running a mixture analysis. We found that heterogeneity was not the cause for the poor performance of the research model. Thus, we moved on to model-generating SEM and ended up with a statistically significant empirical model (root mean square error of approximation [RMSEA] 0.051, Tucker-Lewis index [TLI] 0.906, comparative fit index [CFI] 0.915, and standardized root mean square residual 0.062). According to our empirical model, the statistically significant drivers for behavioral intention were effort expectancy (beta=.191, *P*<.001), self-efficacy (beta=.449, *P*<.001), threat appraisals (beta=.416, *P*<.001), and perceived barriers (beta=−.212, *P*=.009).

**Conclusions:**

Our research highlighted the importance of health-related factors when it comes to eHealth technology adoption in the consumer context. Emphasis should especially be placed on efforts to increase consumers’ self-efficacy in eHealth technology use and in supporting healthy behavior.

## Introduction

### Overview

Constantly increasing health care costs have led countries and health care providers to the point where health care systems must be reinvented. At the same time, technological development has paved the way for new ways to monitor health and well-being and made it possible for societies to start moving health care toward a more personalized and preventive direction. New digital and mobile technologies point to a future in which consumers will be more involved in the management of their health, generating data that will benefit service providers, helping them to create more targeted, preventive, and personalized solutions [1]. Preventive health care aims at decreasing the likelihood of potential illnesses with protection and early detection [2].

Digital and mobile technologies available for consumers—such as pedometers, hearth-rate measurement instruments, and global positioning system–trackers—are empowering consumers to analyze bodily and mental functioning, something that was once the privilege of health professionals [3].

The possibility that consumers would take more responsibility for their own health using preventive services has created a new promise of cheaper, better, and more efficient electronic health (eHealth) tools [4] and health care systems [5]. This has attracted enthusiasm among health providers to move toward data-driven participatory and personalized health care services [1]. Researchers have recently argued that the digitization of information on a massive scale and the digital infrastructures that collect, process, distribute, and utilize this data are allowing radically new combinations of digital and physical components to produce novel eHealth services [6]. However, research has also identified some challenges such as consumers’ skills [4] and their fears of privacy [7], as well as societal, ethical, political, and cultural concerns [8-10].

For individuals to take more responsibility for their health, it is essential to find ways to liberate the health-related data that organizations have in their possession about consumers’ behavior. It is also important to find incentives for consumers to take active actions with this data. As noted in the study by Kim and Park [11], the effective use of collected health-related data is determined by health consumers’ behavioral intention to measure, store, and manage their own data.

The concept of MyData can be defined as a new approach in personal data management and processing that seeks to look at data management from the consumer’s perspective, and look at personal data as a resource that the consumer can access and control [12]. Thus, the aim of the MyData approach is to provide consumers with the practical means to access, obtain, and use datasets containing their personal information such as medical records and data derived from various online services and to encourage the organizations holding personal data to give consumers control over this data [12]. It has also been argued that if consumers had control over their personal data, they would also have better motivation to take care of their health issues [13,14].

To reach their fullest potential and nationwide adoption, it is crucial to understand the consumer perspective on these new health care solutions. A better understanding of health consumers’ intentions and behavior would aid the development and implementation of effective and efficient strategies [11]. Thus, it is a matter of consumer acceptance, which determines the final usage behavior, and a broadening of new health services into the daily lives of private households [15]. According to the study by Venkatesh et al [16], consumer acceptance of a technology is determined by intention to use it, which again leads to the actual use of the technology.

Recently, eHealth has received a great deal of attention in social sciences, in the domain of Internet studies. However, only 3% of these studies focus on the acceptability of eHealth to consumers, making consumers’ subjective evaluations an understudied domain [17]. This study will address this gap by focusing on the acceptance of preventive eHealth services from the consumer point of view. The objective of this study was to investigate what factors influence the consumer’s intention to use MyData-based preventive eHealth services before use.

Technology acceptance is a relatively mature research area and has received plenty of attention in previous research [18]. The most popular theories in the study field have gathered lots of attention, as has the study of eHealth acceptance [19-21]. However, the most popular theories of technology acceptance used in the study of eHealth were originally developed to study technology acceptance in an organizational context, which is why their fit for a consumer preventive eHealth context can be contended [16]. Thus, the research model for this study will be based on the extended version of the original unified theory of acceptance and use of technology (UTAUT) and UTAUT2, developed specifically for the consumer technology acceptance context. Previous research has also shown that it is crucial to apply theories of health behavior to the study of acceptance and use of preventive eHealth services [22-25] because the intention to use preventive eHealth services is similar to the intention to engage in health protective behavior in the sense that both aim to maintain a healthy life [19]. Interactive technologies such as preventive eHealth services that aim for behavioral change and the promotion of healthier lifestyles for individuals will not be successful unless consumers have sufficient motivation to use those systems and take advantage of them [22,26,27].

In this paper, we combine the key factors of UTAUT2 and health behavior theories to our research model and apply the quantitative structural equations modeling (SEM) approach to analyze the relationships between the variables of the framework. Data for the study was collected using a quantitative questionnaire survey. The survey was a part of a large national research program called Digital Health Revolution (DHR), coordinated by the University of Oulu.

The paper is composed as follows. In section 2 we introduce the previous research and theories that provide the basis for our empirical model. Section 3 presents the methodology and empirical results. Section 4 provides a discussion on the managerial and theoretical implications and the limitations of the study, as well as providing some suggestions for future research.

### Theoretical Background

#### Unified Theory of Acceptance and Use of Technology 2

In the study by Venkatesh et al [18], eight theories were compared and tested to form UTAUT. The eight theories were as follows: the theory of reasoned action, the technology acceptance model (TAM), the motivational model, the theory of planned behavior (TPB), combined TAM and TPB, the model of personal computer use, the diffusion of innovations theory, and social cognitive theory (SCT). The main goal for UTAUT was to combine the contributions of the fragmented and mature technology acceptance literature and to form a unified theory to explain the use and acceptance of technology by individuals. UTAUT incorporates the four direct determinants of intention and use behavior that have a significant effect on the use and acceptance of a technology: performance expectancy, effort expectancy, social influence, and facilitating conditions. *Performance expectancy* is defined as the degree to which using a technology will provide benefits to consumers in achieving some goal [16]. Performance expectancy captures the determinants of perceived usefulness, extrinsic motivation, job fit, relative advantages, and outcome expectations from technology acceptance studies [18]. *Effort expectancy* is defined as the degree of ease associated with consumers’ use of a technology [16], and it captures the determinants of perceived ease of use, complexity, and ease of use [18]. *Social influence* is determined as the extent to which consumers perceive that important others (eg, family or friends) believe that they should use a technology [16]. *Social influence* captures the determinants of social factors, subjective norms, and image from the technology acceptance literature [18]. *Facilitating conditions* are defined as consumers’ perceptions of the external resources and infrastructure that support the use of an information and technology system [16,18]. The definition captures the determinations of perceived behavioral control and compatibility [18]. Facilitating conditions are a direct determinant of behavior in UTAUT and are determined as external conditions that help an individual to perform a behavior. In a consumer context, facilitating conditions can vary between different consumers in relation to application vendors, technology generations, and mobile devices. Thus, consumers who perceive better access to the facilitation conditions will have a higher behavioral intention to use a technology [16].

UTAUT has been successfully adapted and tested in a wide range of contexts such as e-learning [28], mobile services [29,30], mobile banking and mp3 player usage [31], and eHealth [24]. Even though widely used, UTAUT has also been criticized because it was only developed and tested to predict technology acceptance in an organizational context [32]. To close this gap, Venkatesh et al [16] updated and extended the original UTAUT to study technology acceptance and use in a consumer mobile technology context and proposed UTAUT2. The new model incorporates three new constructs: hedonic motivation, price value, and habit. *Hedonic motivation* is determined as the enjoyment that an individual perceives from using a technology, *price value* refers to the perceived value that exceeds the monetary cost of using the technology, and *habit* is determined as the extent to which an individual will perform a behavior automatically because of learning [16].

#### Health Behavior Theories

As noted earlier, the intention to use preventive eHealth services is similar to the intention to engage in health protective behavior. It is therefore crucial to apply theories of health behavior to the study of the acceptance and use of preventive eHealth services. Similar findings have been stated by Riley et al [23], who found evidence for the need for health behavior theories to be applied in the development of user-centric eHealth technologies. Additionally, West et al [25] found a similar need in their study of mobile phone diet apps. Three theories of health protective behavior—namely the health belief model (HBM), protection motivation theory, and SCT—have been successfully adapted in a preventive eHealth context by several researchers [11,19,22,24,33].

##### The Health Belief Model

The HBM was developed from social, psychology, and behavioral theories to help understand why individuals do or do not engage in health-related actions. The basic assumption behind the model is that an individual will either choose to engage in a particular health-related action or not based on the desire to avoid an illness and the belief that a particular action will prevent the illness [34].

The original HBM consists of four basic factors that influence an individual’s health motivation and intention to take preventive action: the perceived susceptibility (to a negative health condition), the perceived severity (of a possible negative health condition), the perceived benefits (of a particular action preventing the negative health condition), and the perceived barriers (to taking a preventive health action) [34].

Thus, according to the HBM, for an individual to engage in preventive health behavior, she or he must have an incentive to take the action, feel threatened by current behavioral patterns, and believe that the change will lead to valued outcomes at acceptable costs. The HBM was later extended with a self-efficacy factor by combining it with SCT.

Self-efficacy is determined as the extent to which one believes that one is able to perform a behavior that leads to a valued outcome. The HBM was initially developed to predict the intention to engage in simple health behavior such as one-time immunization or screening tests. Thus, changing lifelong habits such as eating, drinking, or exercising is a far more difficult process and requires the confidence that one is able to make the change before an intervention is possible [35]. The HBM has been successfully adapted to technology acceptance theories in eHealth acceptance research. For example, Lin [36] combined TAM, innovativeness theory, and the HBM to study asthma care mobile services acceptance and found that the combination of these three models significantly improved the predictive value of the mobile health (mHealth) acceptance model.

##### Protection Motivation Theory

Protection motivation theory is a widely used model for disease prevention and health promotion. Originally developed to explain the effects of fear appeals on health attitudes and behavior, the model is very similar to the HBM. It combines similar factors: severity, vulnerability, response cost, response efficacy, and self-efficacy. According to protection motivation theory, the intention to take preventive health action is formed as a result of two cognitive processes: (1) the individual will evaluate the possible threats (considering severity and vulnerability) of getting an illness and compare them with the intrinsic and extrinsic rewards of a certain negative behavior and (2) the individual will evaluate her or his ability to cope with the threat (response efficacy, self-efficacy, and response cost). As a result of the two processes, the protection motivation will be formulated, which again acts as a force to formulate the intention to take the action [37]. Protection motivation theory has been successfully combined with the technology acceptance theories such as UTAUT in prior research to study mHealth service acceptance [24]. In addition, it has been found to account well for the intention to change one’s behavior [38], which is also why it fits so well for the context of preventive eHealth services that promote behavioral change for a healthier lifestyle.

##### Social Cognitive Theory

SCT has been successfully adapted to the study of the intention to engage in health protective behavior. According to SCT, six basic determinants influence health-related behavior: knowledge of the risks and benefits of health-related actions, perceived self-efficacy, outcome expectations about the costs and benefits of health-related habits, individual goals, and the perceived sociostructural barriers to and facilitators for an individual making the change and achieve her or his goals. Self-efficacy is the central part of SCT in that it influences behavior both directly and via other determinants. According to Bandura [22], other theories of health behavior predict an individual’s health habits well, but SCT is the only theory to provide predictors and principles to guide individuals in behavioral change.

### The Research Model

Due to the objective of this study to investigate MyData-based preventive eHealth service acceptance in a consumer context, UTAUT2 will be adapted as the basis for our research model. As most organizations do not yet provide data for individuals in a format that would be useful and practical from the viewpoint of health data analytics and new health services [39], the use behavior and price value originally presented in UTAUT2 will be excluded from this model. Use behavior will be excluded because the target group of the study cannot experience the use situation of the MyData-based preventive eHealth services. The price value, on the other hand, will be excluded as no service models or price structures for the services in question were yet developed at the time of writing this study.

**Figure 1:**
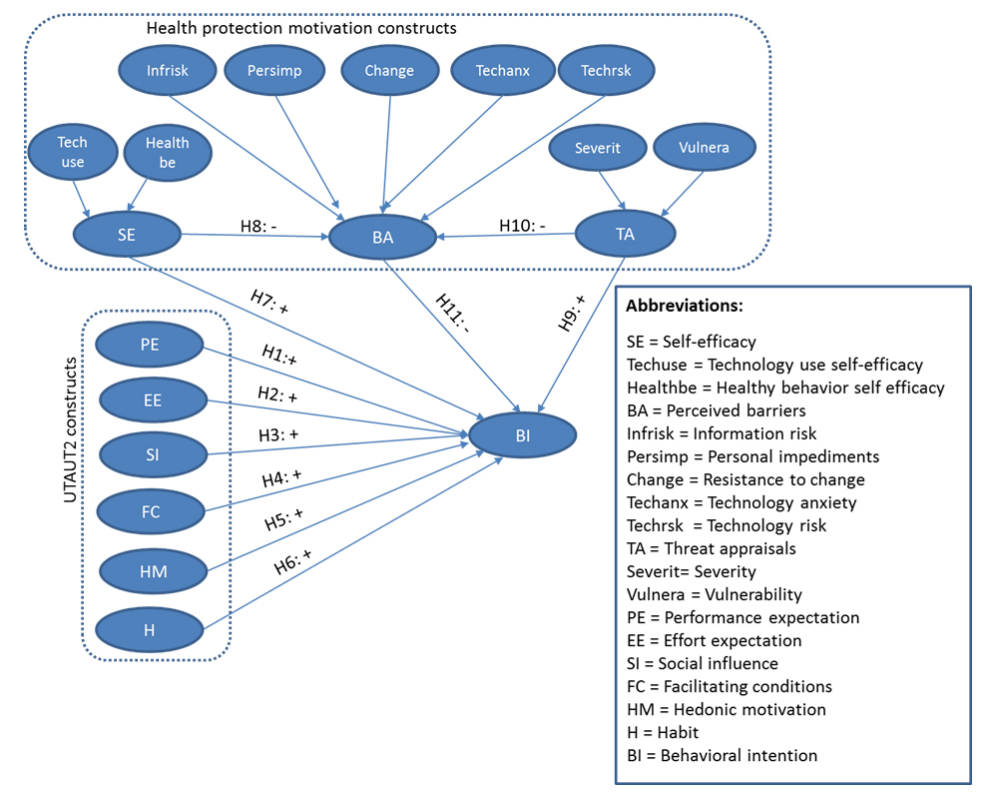
The proposed model for consumer acceptance of future MyData-based electronic health (eHealth) services.

To take account of health behavior factors, the three health behavior theories that are considered complement the UTAUT2 model with three health protection motivation constructs: self-efficacy, threat appraisals, and perceived barriers (see Figure 1). Although excluded from UTAUT as an insignificant factor for technology acceptance in an organizational context [18], self-efficacy is an important factor in health behavior theories, as it explains how an individual believes in her or his ability to achieve health outcomes [22,34,37]. Previous research has also shown that both technology-related self-efficacy factors [19,24] and healthy behavior–related self-efficacy factors [28] are significant factors in promoting health behavior [40]. Therefore, in our research model, self-efficacy is composed of two elements: technology use and healthy behavior.

Threat appraisals on the other hand play an important role in creating the motivation to take action to avoid a negative health outcome or to improve one’s poor health condition [37] while using preventive eHealth services. According to protection motivation theory, threat appraisals include two components: severity (the seriousness of a possible health threat) and an individual’s vulnerability (the risk of encountering a health threat). Both components have been found significant in previous research [19,24,36]. Hence, in our research model, we also apply the two-component construct for threat appraisals.

Finally, a wide variety of perceived barriers can have a significant negative influence on behavioral intention to use those systems. In the acceptance of new technologies, consumers have been found to have perceived psychological risks that prevent the adoption of new technologies [25]. There is evidence that consumers generate perceived risks regarding preventive eHealth services, especially before use [33]. Relevant issues that have been considered as barriers to the acceptance of preventive eHealth services are, for example, concerns about information abuse, privacy invasion, personal impediments [19], resistance to change, and technological anxiety [20]. Negative perceptions of the quality of technology-based services can also influence the acceptance of those services [41]. Preventive eHealth services are new innovations that require individuals to gather and store personal and sensitive health data in personal health records [11]. In addition, the adoption of preventive eHealth does not only include adopting a new technology but can also aim for significant changes in an individual’s lifestyle [42]. To capture the effect of potential barriers as widely as possible, our research model includes five barrier components considered in previous research: information risk, personal impediments, resistance to change, technological anxiety, and technology risk. A summary table of the studies used as a reference to our research is included in Multimedia Appendix 1.

#### Research Hypotheses

##### Performance Expectancy

In the context of preventive eHealth services, the use of the technology will provide benefits for an individual in preventing her or him from falling ill [43]. For example, in the future, the analysis and visualization of personal data will allow individuals to understand their health better and to support and enable self-care [39]. Performance expectancy has been found to be the most important direct predictor of behavioral intention in most information technology acceptance [18] and preventive eHealth studies [19,24,44].

Hypothesis 1: Performance expectancy will influence behavioral intention positively.

##### Effort Expectancy

Effort expectancy has been found to have a significant effect, especially for the elderly consumers’ acceptance of preventive eHealth services [24]. On the other hand, Jung and Loria [45] found that the difficulty associated with the use of preventive eHealth technology is usually related to the user’s lack of experience with the use of the Internet in general. Similar findings have been presented by Jung and Berthon [46] about the difficulty of using preventive eHealth services. Effort expectancy has been found to be positively associated with the behavioral intention to use a technology in technology acceptance literature [18].

Hypothesis 2: Effort expectancy will influence behavioral intention positively.

##### Social Influence

Social influence can occur as information about the benefits of using preventive eHealth services from a health professional’s advice or through media education and also as encouragement from friends or relatives. Social influences can also provide a reminder or trigger for a motivated person to take action. Social influence is determined here as encouragement or reminders from important others or media channels to promote the use of preventive eHealth services [16,36]. Social influence has been found to significantly influence behavioral intention to use a technology [18,19,36].

Hypothesis 3: Social influence will influence behavioral intention positively.

##### Facilitating Conditions

The ease of access to the preventive eHealth services is an important factor, especially in the early stages of adoption, before the quality of the information has been determined [46]. The significance of assistance, education, and guidelines has been noted in the study of preventive eHealth services because they help service providers to increase users’ comfort and confidence in using the system. In addition, the compatibility of preventive eHealth services with mobile phones and other popular devices that are commonly used by consumers could improve the wider adoption of eHealth services [33].

Facilitating conditions have been found to have a significant positive effect on consumers’ behavioral intention to use mobile technologies [16]. Thus, consumers who perceive the future MyData-based preventive eHealth services as both easily accessible from anywhere at any time and compatible with their devices are more willing to use these services.

Hypothesis 4: Facilitating conditions will influence behavioral intention positively.

##### Hedonic Motivation

Hedonic motivation is determined as the fun or pleasure that a consumer derives from using a technology [16]. Other factors from technology acceptance literature that deal with similar emotions (such as enjoyment, joy, or liking) and can be incorporated under the determination of hedonic motivation are the consumer’s attitude toward a behavior and intrinsic motivation [18]. Hedonic motivation has been found to strongly impact on behavioral intention to use a technology in a consumer context [16,26].

Hypothesis 5: Hedonic motivation will influence behavioral intention positively.

##### Habit

Habit is determined as the extent to which an individual believes performing a behavior (eg, using mobile apps to track exercise) to be automatic as a result of learning during past behavior [47]. According to Venkatesh et al [16], the repeated performance of a behavior in a similar context or environment can produce stored intentions and positive attitudes that are associated with the behavior. Thus, if the person faces a similar context or environment again to that in which the habit was formulated, the stored intentions can be triggered that lead to the same behavior [16].

Hypothesis 6: Habit will influence behavioral intention positively.

##### Self-Efficacy

Self-efficacy is defined as one’s confidence in one’s ability to successfully perform a behavior that leads to a valued outcome. The definition overlaps with the perceived behavioral control factor in the TPB. Self-efficacy is an important factor because it influences an individual’s aspirations and goals in general. If one has high self-efficacy, one will set higher goals and will have higher expectations of achieving those goals [22]. Consumers with high self-efficacy will learn faster and be more confident to use preventive eHealth services, which positively influences acceptance of those technologies [24]. As preventive eHealth services are promoting significant changes in consumers’ lifestyles that demand individual effort, self-efficacy influences both the acceptance of those goals and success in achieving them. In addition to using preventive eHealth services successfully and achieving these changes in lifestyle, one must have the ability to first, use the technology, and second, one must be able to comply with the healthy behavior.

Thus, a person with high self-efficacy will be likely to believe that using preventive eHealth services will generate better health outcomes compared with a person with low self-efficacy.

Following this, the following can be hypothesized:

Hypothesis 7: Self-efficacy will influence behavioral intention positively.

Another significant aspect of self-efficacy is that it negatively influences cognitive barriers. If one has high efficacy, one will view obstacles as surmountable and will continue on the path to achieving one’s goals [22]. An individual is likely to face some obstacles while trying to improve her or his health. According to protection motivation theory, an individual will create the motivation to take part in a preventive health action based on her or his evaluation of the intrinsic and extrinsic rewards of negative behavior (such as unhealthy eating habits or watching TV instead of doing exercise) weighed against the threat of reducing her or his health status. Here, the intrinsic rewards (bodily pleasure) and extrinsic rewards (peer approval) act as barriers to preventive health behavior [37]. Thus, the higher self-efficacy of an individual will negatively influence perceived barriers, as the individual will see those barriers as surmountable with self-control and will also be more involved in achieving her or his goals [22].

Hypothesis 8: Self-efficacy will influence perceived barriers negatively.

##### Threat Appraisals

The stronger the threat appraisals are, the stronger the motivation for an individual to take part in healthy behavior [34]. According to Wilkowska and Ziefle [15], consumers with a higher need for health care and higher threat appraisals, such as chronically ill patients, also tend to pay less attention to the risk factors related to eHealth services. Thus, it can be expected that consumers with higher threat appraisals will have a stronger behavioral intention to take part in preventive health behavior and will have a stronger behavioral intention and fewer barriers to using preventive eHealth services. In addition, Kim and Park [11] also found that persons who perceive high health threats will also perceive preventive eHealth services as more useful than healthy people. Following these assumptions, the following can be hypothesized:

Hypothesis 9: Threat appraisals will influence behavioral intention positively.

Hypothesis 10: Threat appraisals will have an influence on the perceived barriers negatively.

##### Perceived Barriers

Perceived barriers are defined in the HBM as potential negative aspects that would be expensive, dangerous, unpleasant, inconvenient, or time-consuming when taking a particular health action [34]. Relevant issues related to information risks and technology risks that cause significant barriers to the acceptance of preventive eHealth services are, for example, worrying about information abuse, privacy invasion, the lack of precision of equipment, and excessive charges [19]. The study by Guo et al [20] found that among elderly users, resistance to changing their lifestyle and technology anxiety regarding eHealth technologies both produce significant cognitive barriers to technology acceptance. Other issues that can raise concerns in consumers are, for example, how and for what purpose all the health data gathered from them will be used by the service provider [48].

According to the HBM, barriers have a significant effect on an individual’s intention to take health protective actions [34]. Thus, consumers who perceive barriers to health behavior and the use of preventive eHealth technologies will have less behavioral intention to use MyData-based preventive eHealth services. On the basis of the above, the following can be hypothesized:

Hypothesis 11: Perceived barriers influence behavioral intention negatively.

## Methods

### Measurement

The research model is composed of 16 constructs. The constructs were measured with multiple, reflective items on a 5-point Likert scale. Most of the measurement items were adapted from prior research to preserve content validity. The only exception is the construct *performance expectancy*, in which three additional items were included to reflect the expected performance of the services under the study. However, items were prior evaluated by medical, business, and information systems researchers from the DHR project and statistically tested similarly to all the other items in the other factors. Because at the time of the survey the studied service did not yet exist, we had to measure habit with items reflecting the use of existing eHealth or wellness technologies such as heart rate monitors and mobile phone apps related to exercise or nutrition. This approach is justified by Limayem et al [47], who define habit as the degree to which consumers use technologies automatically because of learning. They further state that habit is conditioned by stable contexts that are characterized by the presence of similar situational cues and goals across more or less regularly occurring situations and that the strength of habit related to multifunctional technologies (such as using different eHealth apps) depends on the degree of frequency and diversity of prior use of these types of technologies. Further justification can be found in the study by Venkatesh et al [16], which found that a repeated performance in a similar context can result in habit formation. Hence, we can assume that because the existing eHealth and wellness services can be regarded quite similar to the developed MyData-based eHealth services (the main differentiator is extent of the utilization of personal data in personalizing the service), the extent of the use of the existing eHealth can be used in operationalizing the habit construct. The original items in the survey instrument were translated into Finnish and reviewed by four experienced researchers from the University of Oulu. A back translation by a professional translator was also performed to ensure item content validity. The items are included in Multimedia Appendix 2.

### Data Collection

Data for the study was collected using a quantitative, Web-based questionnaire survey. The survey was a part of a large national research program, DHR, coordinated by the University of Oulu. The goal of this multidisciplinary research program is to enable the utilization of data about the individual as part of personal, preventive services, which in turn will improve citizens’ opportunities for self-management of their well-being. The aim of this study was to investigate what factors influence consumers’ intentions to use a MyData-based preventive eHealth service before use. The sample frame was the faculty and the staff of the University of Oulu, a total of 2852 people. The link to the survey was sent via email. The email included both the link to the Web-based survey in WebPropol survey service and a cover letter explaining the research phenomenon and research context, the purpose of the survey, and the use of the data, as well as encouragement to answer. The email was sent to the sample frame in March 2015, and the link for the survey was open for 1 week. We had a total of 855 respondents out of 2852 (29.98% response rate) who voluntarily chose to answer the survey. The demographic distribution of the respondent group is shown in Table 1.

The gender distribution of the respondent group was female dominated with over two-thirds of the respondents being women. Almost 70% (579/855) of the respondents belonged to the two youngest age groups.

### Empirical Analyses

Empirical analyses were made using SEM with Mplus software, version 7.4. Estimations were made using maximum likelihood based on covariance matrix. First, we ran traditional SEM for the research model. That was not statistically significant, and thus, we tested possible heterogeneity in the data by running mixture analysis.

We found that heterogeneity of the data was not an issue in this case. Thus, we moved on toward model-generating SEM [49]. On the basis of theoretical justifications and fit indices of the generated model (root mean square error of approximation [RMSEA] 0.051—according to Browne and Cudeck [50], a RMSEA value below 0.8 stands for a reasonable error of approximation; Tucker-Lewis index [TLI] 0.906 and comparative fit index [CFI] 0.915—Hu and Bentler [51] argue that a value close to 0.95 for both TLI [nonnormed fit index] and CFI are needed before we can conclude that there is a relatively good fit between the hypothesized model and the observed data; and standardized root mean square residual 0.062), we judge the empirical model to be statistically significant. Average variances extracted (AVEs), squared AVEs, and composite reliabilities (CRs) are presented in Table 2. According to Hair et al [52], all factors excluding the *Healthbe* and CR of *Techanx* are statistically significant. However, according to Steenkamp and van Trijp [53], all factors fulfill a weak and stronger convergent validity because factor loadings are statistically significant and coefficients are substantial.

**Table 1:** The demographic distribution of the respondents.

Characteristics		n (%)
**Gender**		
	Male	305 (35.7)
	Female	550 (64.3)
	Total	855 (100)
**Age (years)**		
	18-25	352 (41.2)
	26-35	227 (26.5)
	36-45	119 (13.9)
	46-55	107 (12.5)
	56-65	48 (5.6)
	66 and over	2 (0.2)
	Total	855 (100)

**Table 2:** Construct reliabilities.

Label	Infrisk^a^	Persimp^b^	Change^c^	Techanx^d^	Techrsk^e^	Severit^f^	Vulnera^g^	Techuse^h^	Healthbe^i^	EE^j^	BI^k^
AVE^l^	0.669	0.590	0.707	0.508	0.592	0.718	0.528	0.655	0.488	0.692	0.684
Squared AVE	0.820	0.768	0.841	0.713	0.769	0.847	0.762	0.809	0.699	0.832	0.827
CR^m^	0.852	0.733	0.853	0.679	0.736	0.944	0.637	0.838	0.582	0.833	0.885

^a^Information risk.

^b^Personal impediments.

^c^Resistance to change.

^d^Technology anxiety.

^e^Technology risk.

^f^Severity.

^g^Vulnerability.

^h^Technology use self-efficacy.

^i^Healthy behavior self-efficacy.

^j^Effort expectancy.

^k^Behavioral intention.

^l^AVE: average variance extracted.

^m^CR: composite reliability.

**Figure 2:**
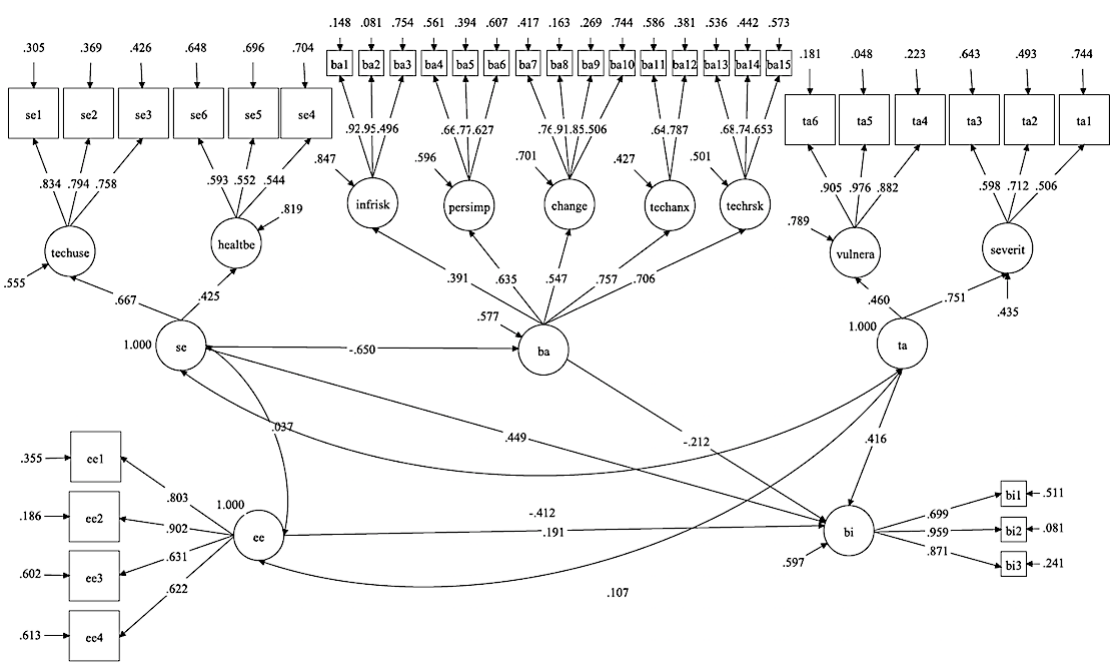
A model-generated structural equation model.

**Table 3:** Structural equation model; beta and *P* values.

Predicted construct and predictor constructs		Beta	*P* value
**Behavioral intention**			
	Effort expectancy	.191	<.001
	Self-efficacy	.449	<.001
	Threat appraisals	.416	<.001
	Perceived barriers	−.212	.009
**Perceived barriers**			
	Self-efficacy	−.650	<.001

## Results

The hypotheses and their judgments based on the SEM results are shown in Table 4. Quite surprisingly, most of the hypotheses related to explanatory UTAUT2 constructs were rejected. Only effort expectancy was found to have a statistically significant effect on behavioral intention. The results confirm the health protection motivation constructs of our research model. On the basis of the empirical model, information risk, personal impediments, resistance to change, technological anxiety, and technological risk are the significant dimensions of barriers. Severity and vulnerability are dimensions of threat appraisals, and finally, technology use and healthy behavior are dimensions of self-efficacy. All three health motivation constructs have a significant effect on behavioral intention. Furthermore, our model also found a correlation between self-efficacy and threat appraisals. In addition, barriers seem to have a negative mediating effect from self-efficacy toward behavioral intention.

**Table 4:** The tested hypotheses.

Hypothesis (H)	Description	Judgment
H1	Performance expectancy will influence behavioral intention positively.	Rejected
H2	Effort expectancy will influence behavioral intention positively.	Accepted
H3	Social influence will influence behavioral intention positively.	Rejected
H4	Facilitating conditions will influence behavioral intention positively.	Rejected
H5	Hedonic motivation will influence behavioral intention positively.	Rejected
H6	Habit will influence behavioral intention positively.	Rejected
H7	Self-efficacy will influence behavioral intention positively.	Accepted
H8	Self-efficacy will influence perceived barriers negatively.	Accepted
H9	Threat appraisals will influence behavioral intention positively.	Accepted
H10	Threat appraisals will influence perceived barriers negatively.	Rejected
H11	Perceived barriers will influence behavioral intention negatively.	Accepted

## Discussion

### Principal Findings

Contrary to prior research applying UTAUT in an eHealth context [54,55], performance expectancy was not found to be a significant factor in explaining behavioral intention. One reason for this result could be that our research was conducted among consumers, whereas the majority of the existing research considers the factors influencing technology use by health care professionals. All in all, most of the hypotheses related to UTAUT2 constructs were rejected. Of the UTAUT2 constructs included in the research model, only effort expectancy seemed to have a significant influence on behavioral intention. It thus seems that the model does not perform well in explaining consumers’ intentions to use eHealth services before actual use, which was the case in this study.

Regarding the health protection motivation constructs, the results were the opposite as all three constructs—threat appraisals, self-efficacy, and perceived barriers—were found to have a significant influence on behavioral intention. In addition to the fact that our study focused on the intention to use future services instead of actual service use, there are other possible reasons for the poor performance of UTAUT2 constructs. Due to the focus on preventive eHealth services, the respondent group in our study had a proportion of healthy people who did not have a chronic illness. Our study also had younger participants compared with many other studies [56,57]. Furthermore, whereas many other studies focus on the usage of eHealth services as such [56,58], our study focused on the situations in which a person gave permission to use her or his data in eHealth services.

### Theoretical Implications

Our study contributes to the health information technology literature in two ways. First, we provide a research model that combines the standard UTAUT model with health protection motivation constructs, thus bridging the gap between technology adoption and health behavior theories. Second, our study focuses on increasing understanding of the factors influencing consumers’ eHealth technology acceptance instead of focusing on the acceptance of health care professionals.

### Managerial Implications

Our research highlights the importance of the two health protection motivation constructs. Both threat appraisals and self-efficacy were found to be significant determinants of the intention to use future preventive eHealth services. Both technology use self-efficacy and healthy behavior self-efficacy were found to be significant components of the construct. This result suggests that to promote the use of new preventive eHealth services, emphasis must be placed on both technology use and healthy behavior education. Increased awareness of eHealth technology use and healthy behavior will also have a positive effect as it will diminish the effects of perceived barriers, which were found to have a significant negative impact on intention to use a service.

Typically, the importance of effort expectancy has been found in the studies on elderly people’s intention to use eHealth services [58]. Our result is thus surprising, as the participants in our study were mainly younger people (under 55 years). The fact that effort expectancy was found as a significant determinant of intention to use the service implies the importance of design issues in new eHealth services adoption. The results by Daim et al [59], indicating that different levels of technology understanding and health literacy can have a significant influence on the user experience of preventive eHealth services, further stresses the importance of design issues. The understanding of the importance of effort expectancy and self-efficacy related to the MyData-based preventive eHealth services has a significant impact on understanding the adoption of the MyData approach and MyData-based eHealth services in general.

### Limitations

There are also some limitations that must be considered when considering the generalizability of our results. The research context of future technologies and services that have not been developed yet to their fullest potential poses some limitations. First, the quantitative survey was based on a hypothetical use situation, and thus, the target group did not experience the actual use of the service. Second, the construct of *habit* had to be measured based on the use experience of existing eHealth apps that are only possibly related to the MyData-based preventive eHealth services of the future. Another limitation for this study is the sampling frame of university staff and faculty. Even though the survey link was sent to people with different educational backgrounds and work descriptions, we must however acknowledge that so called “blue-collar” workers may be underrepresented. Furthermore, in our study, women are overrepresented because 63.4% (550/855) of the respondents were women and their share of Finnish working age people is 49.65% [60]. This may cause bias because women have been found to be more likely to engage in eHealth activities [61]. Poor performance of UTAUT constructs could also be considered as a limitation. Our approach was not to test the performance of the UTAUT2 model but to find as holistic a model as possible with statistical support, so we included both UTAUT2 and health protection motivation constructs to the research model. This research model, together with the fact that we were studying the intention to use future services, may all play a role in the results. However, based on Model Generating Structural Equation Modeling (MGSEM), we, for example, removed factor habit, which is similar in result with Oliveira et al [62]. Furthermore, our results give rise to similar conclusions to previous studies, which highlight the importance of increasing eHealth-related self-efficacy through education and making services easy to use [63,64]. Thus, we can argue that our limitations may not be too limiting because our results are in line with previous studies.

The above limitations lead us to the following suggestions for future research avenues. First, future research should test the acceptance of these preventive MyData-based eHealth services in an actual-use context and investigate how the direct factors perform in that context. Second, future research should also consider a wider coverage of consumers with a nonacademic background to see the relationships between the considered variables in a larger demographic setting. The importance of inclusion of different demographic segments in eHealth research and promotion activities was also stressed in the study by Kontos et al [61].

### Conclusions

Our study contributes to the exploration of the factors influencing the consumers’ intention to use MyData-based preventive eHealth services before use. We combine the key factors of UTAUT2 and health behavior theories to our research model and apply the quantitative SEM approach to analyze the relationships between the constructs of the research model. Our results indicate that UTAUT2 constructs perform poorly. Only effort expectancy had a significant effect on the intention to use. Contrary to UTAUT2 constructs, all constructs adapted from health behavior theories—threat appraisals, self-efficacy and perceived barriers—were found to have a significant effect on the intention to use. These results suggest that to promote the adoption of preventive eHealth services among consumers, it is essential to invest in increasing the general awareness of healthy behavior and in the expertise of using eHealth technologies. From societal perspective, this implies increasing investment in health and technology-related education. From service perspective, higher probability of new preventive eHealth service adoption could be achieved, for example, by increasing consumer involvement in the creation of services through collaborative practices.

## Appendix

Multimedia Appendix 1 Summary table of eHealth acceptance studies.

Multimedia Appendix 2 Questionnaire items.

## Abbreviations

AVE: average variance extracted
CFI: comparative fit index
CR: composite reliability
DHR: Digital Health Revolution
eHealth: electronic health
HBM: health belief model
mHealth: mobile health
RMSEA: root mean square error of approximation
SCT: social cognitive theory
SEM: structural equations modeling
TAM: technology acceptance model
TLI: Tucker-Lewis index
TPB: theory of planned behavior
UTAUT: Unified theory of acceptance and use of technology
