# Supplementary material for: Consumer Adoption of Future MyData-Based Preventive eHealth Services: An Acceptance Model and Survey Study
Source: J Med Internet Res. 2017 Dec 22;19(12):e429. doi: 10.2196/jmir.7821 (PMC5756317; doi:10.2196/jmir.7821)
Supplement: Multimedia Appendix 1 [file jmir_v19i12e429_app1.pdf]

Reference	Theory	Dependent variable	Main findings
[11]	TAM, HIT	Behavioral intention	Perceived threat, perceived usefulness, and perceived ease of use significantly affected health consumers' attitude and behavioral intention
[15]	Modified UTAUT	Perceived usefulness	Security and privacy aspects play an important role for acceptance and usage of medical assistive technologies.
[16]	UTAUT/ UTAUT2	Behavioral intention, Use behavior	Confirmation of the important roles of hedonic motivation, price value, and habit in influencing technology use and in UTAUT2
[19]	TAM, HBM	User adoption	Perceived usefulness and benefits, perceived barriers and external cues positively affect user attitude. Perceived service availability significantly influences the perceived ease of use, perceived usefulness and benefits, which with attitude directly enhances intention.
[20]	TAM	Health service Adoption behavior	Resistance to change influences perceived usefulness; Technology anxiety associated negatively with perceived ease of use but positively with resistance to change; Dispositional resistance to change associated negatively with perceived ease of use but positively with resistance to change.
[21]	UTAUT	Behavioral intention	Facilitating factors have effects on the behavioral intention through the performance and effort expectancy. Performance expectancy, effort expectancy and social influence have positive effects on behavioral intentions.
[22]	Social cognitive theory	Human motivation/ behavior	Core belief affects each of the basic processes of personal change
[24]	TAM, TPB UTAUT, PMT	Adoption intention	Most influential factors: response efficacy, subjective norm, self-efficacy and perceived ease of use. Subjective norm and perceived ease of use have significant impacts on adoption intention.
[26]	TAM	Health behavior	Perceived risk affects use of the information technology service negatively. Intrinsic and extrinsic motivation have a positive influence on intention to use the service and perceived risk has a negative influence.
[27]	Social survey	Public attitudes and perceptions	Consumers whose physicians use EHR were more likely to believe that EHR and HIE will improve healthcare when compared to others. Experience with a physician using an EHR had no relationship with privacy concerns.
[33]	UTAUT	Behavioral intention	Users have a more positive perception of service benefits than non-users. Both had a high negative perception of service risk. Users also had a more positive perception of intimacy and communication associated with the services than non-users. Both users and non-users had a high behavioral intention to use the services.
[36]	TAM, HBM	Use intention	User's attitude is most critical determinant of their intention, followed by perceived usefulness, perceived ease-of-use, external cues to actions, and innovativeness.
[39]	Qualitative inquiry	Behavioral & Use intention	The usefulness of the acquired personal health data affects intention. More emphasis should be placed on providing the data in a reusable form.
[41]	UTAUT	Behavioral	Perceived risk, technology type, and gender were

		intention	significant moderating variables. The effects of user experience were marginal after the variance of errors were removed.
[42]	Review	PHR adaption, Consumer behavior	Barriers to PHR adoption among patients include cost, privacy concerns, inconvenience, design shortcomings, and the inability to share information across organizations.
[43]	TAM	Adoption of personal health management services	Perceived ease of use and perceived usefulness, significantly affected attitude. Perceived ease of use significantly affected perceived usefulness.
[44]	IT acceptance model	Patient acceptance of eHealth services	Antecedent factors of satisfaction with provider, information-seeking preference, and Internet dependence uniquely predicted constructs in the models.
[45]	TAM	Patient acceptance of / Use intention of eHealth services	Usefulness and perceived ease of use are the main determinants of people's intention to use the service. Additional factors critical to the acceptance are the importance of the compatibility of the services with citizens' needs and trust in the service provider.
[46]	The e-health acceptance model	Behavioral intention	A new TAM –based theoretical model, new included concepts are compatibility, credibility, perceived risk and perceived accessibility
