# Supplementary material for: Consumer Adoption of Future MyData-Based Preventive eHealth Services: An Acceptance Model and Survey Study
Source: J Med Internet Res. 2017 Dec 22;19(12):e429. doi: 10.2196/jmir.7821 (PMC5756317; doi:10.2196/jmir.7821)
Supplement: Multimedia Appendix 2 [file jmir_v19i12e429_app2.pdf]

Construct items:	Item source:
**Performance Expectancy (PE)**	
PE1: The service helps one to the monitor his/her health.	Lin 2011
PE2: The service is helpful in managing one's health data.	Deng 2013
PE3: The services helps to utilize one's own health data.	New item
PE4: The services gives feedback on how behavior affects health.	New item
PE5: The services educate one to affect his/her own health.	New item
**Effort Expectancy (EE)**	
EE1: Learning how to use the service is easy for me.	Venkatesh, Thong and Xu 2012
EE2: Interaction with the service is clear and understandable.	Venkatesh, Thong and Xu 2012
EE3: I can get the services to do what I want them to do.	Sun, Wang, Guo and Peng 2013
EE4: Instructions for using the service will be easy to follow.	Hung and Jen 2010
**Social Influence (SI)**	
SI1: People who are important to me think that I should use the service	Venkatesh, Thong and Xu 2012
SI2: People who influence my behavior think that I should use the service.	Venkatesh, Thong and Xu 2012
SI3: People whose opinions I value prefer that I use the service.	Venkatesh, Thong and Xu 2012
SI4: Before the use situation, I have learn about the importance<br>of these services from TV broadcast, newspaper, and other media.	Lin 2011
**Facilitating Conditions (FC)**	
FC1: The services are compatible with other technologies I use.	Venkatesh, Thong and Xu 2012
FC2: I can get help from others when I have difficulties using the service. (changed to EE)	Venkatesh, Thong and Xu 2012
FC3: I would be able to use the service at any time, from anywhere.	Deng 2013
FC4: Using the service fits into my life-style.	Moore and Benbasat 1991
FC5: Using the service fits well with the way I like to do thing	Moore and Benbasat 1991
**Hedonic Motivation (HM)**	
HM1: Using the service is fun.	Venkatesh, Thong and Xu 2012
HM2: Using the service is enjoyable.	Venkatesh, Thong and Xu 2012
HM3: Using the service is very entertaining.	Venkatesh, Thong and Xu 2012
**Habit (H)**	
H1: The use of this technology/service has become a habit for me	Venkatesh, Thong and Xu 2012
H2: I am addicted to using this technology/service	Venkatesh, Thong and Xu 2012
H3: I must use this technology/service	Venkatesh, Thong and Xu 2012
H4: Using this technology/serviceto manage my health is an obvious choise for me	Venkatesh, Thong and Xu 2012
**Self-Efficacy (SE)**	
**Tehcnology use (set)**	
set1: I would be confident in using preventive eHealth services even if there was no one around to show me how to use them.	Deng 2013
set2: I feel confident managing my health with the preventive eHealth services.	Deng 2013
set3: I have the skills necessary to learn and use the preventive eHealth services.	Sun, Wang, Guo and Peng 2013
**Healthy behavior (seh)**	
seh1: It is easy for me to cut back on unhealthy treats.	Moorman and Matulich 1993
seh2: It is easy for me to eat fresh fruits and vegetables regularly.	Moorman and Matulich 1993
seh3: It's easy for me to exercise three days a week.	Moorman and Matulich 1993
**Threat Appraisals (TA)**	
**Vulnerability (tav)**	
tav1: I find that I can fall ill easier than others.	Deng 2013
tav2: I find that my health is getting poor than times before.	Lin 2011
tav3: I find that I can suffer from high blood pressure, diabetes heart disease and other chronic diseases in the future.	Deng 2013
**Severity (tas)**	
tas1: If I suffered the stated problems, it would be severe.	Sun, Wang, Guo and Peng 2013
tas2: If I suffered the stated problems, it would be serious.	Sun, Wang, Guo and Peng 2013
tas3: If I suffered the stated problems, it would be significant.	Sun, Wang, Guo and Peng 2013
**Barriers (BA)**	
**information risk (bai)**	
bai1: I am concerned that the services are not adequately secure and that they might lead to the leak or abuse of my personal information.	Deng 2013
bai2: I am concerned that the services would violate my privacy.	Deng 2013
bai3: I am concerned that the reliability of health information provided by	Deng 2013
**Personal impediments (bap)**	
bap1: I am concerned that I might forget to use the service.	Deng 2013
bap2: I am concerned that I might be too busy to use the service.	Deng 2013
bap3: I have to spend effort on learning how to use the services.	Sun, Wang, Guo and Peng 2013
**Resistance to change (bar)**	
bar1: I don't want the services to change the way I currently live.	Guo, Sun, Wang, Peng and Yan 2013
bar2: I don't want the services to change the way I deal with health-relevant problems.	Guo, Sun, Wang, Peng and Yan 2013
bar3: I don't want the services to change the way I keep myself healthy.	Guo, Sun, Wang, Peng and Yan 2013
bar4: I don't want the services to change the way I interact with other people.	Guo, Sun, Wang, Peng and Yan 2013
**Technology anxiety (bata)**	
bata1: I hesitate to use technology for fear of making mistakes I cannot correct.	Guo, Sun, Wang, Peng and Yan 2013
bata2: Mobile health services are intimidating to me. (changed to SE)	Guo, Sun, Wang, Peng and Yan 2013
**Technology risk (batr)**	
batr1: It is probable that these services would frustrate me because of their poor performance.	Im, Kim and Han 2008
batr2: Comparing with other technologies, these services have more uncertainties.	Im, Kim and Han 2008
batr3: It is uncertain whether the service would be as effective as I think.	Im, Kim and Han 2008
**Behavioral intention (BI)**	
BI1: If necessary, I would use the service.	Lin 2011
BI2: If available, I would use the service in the future.	Koivumäki, Ristola and Kesti 2008
BI3: As a whole, I am highly willing to use the service	Lin 2011
